# Identification and characterization of the *GmRD26* soybean promoter in response to abiotic stresses: potential tool for biotechnological application

**DOI:** 10.1186/s12896-019-0561-3

**Published:** 2019-11-20

**Authors:** Elinea O. Freitas, Bruno P. Melo, Isabela T. Lourenço-Tessutti, Fabrício B. M. Arraes, Regina M. Amorim, Maria E. Lisei-de-Sá, Julia A. Costa, Ana G. B. Leite, Muhammad Faheem, Márcio A. Ferreira, Carolina V. Morgante, Elizabeth P. B. Fontes, Maria F. Grossi-de-Sa

**Affiliations:** 1 Embrapa Genetic Resources and Biotechnology, Brasília, DF Brazil; 2 Federal University of Brasília, Brasília, DF Brazil; 3 Federal University of Viçosa, Viçosa, MG Brazil; 4 Federal University of Rio Grande do Sul, Porto Alegre, RS Brazil; 5 Agricultural Research Company of Minas Gerais State, Uberaba, MG Brazil; 6 Catholic University of Brasilia - Post-Graduation Program in Genomic Sciences and Biotechnology, Brasília, DF Brazil; 7 National University of Medical Sciences, Rawalpindi, Punjab Pakistan; 8 Federal University of Rio de Janeiro, Rio Janeiro, RJ Brazil; 9 Embrapa Semi-Arid, Petrolina, PE Brazil

**Keywords:** Stress-responsive promoter, Drought tolerance, Abscisic acid, Promoter modules analysis, Gene-promoter characterization

## Abstract

**Background:**

Drought is one of the most harmful abiotic stresses for plants, leading to reduced productivity of several economically important crops and, consequently, considerable losses in the agricultural sector. When plants are exposed to stressful conditions, such as drought and high salinity, they modulate the expression of genes that lead to developmental, biochemical, and physiological changes, which help to overcome the deleterious effects of adverse circumstances. Thus, the search for new specific gene promoter sequences has proved to be a powerful biotechnological strategy to control the expression of key genes involved in water deprivation or multiple stress responses.

**Results:**

This study aimed to identify and characterize the *GmRD26* promoter (p*GmRD26*), which is involved in the regulation of plant responses to drought stress. The expression profile of the *GmRD26* gene was investigated by qRT-PCR under normal and stress conditions in Williams 82, BR16 and Embrapa48 soybean-cultivars. Our data confirm that *GmRD26* is induced under water deficit with different induction folds between analyzed cultivars, which display different genetic background and physiological behaviour under drought. The characterization of the *GmRD26* promoter was performed under simulated stress conditions with abscisic acid (ABA), polyethylene glycol (PEG) and drought (air dry) on *A. thaliana* plants containing the complete construct of p*GmRD26*::*GUS* (2.054 bp) and two promoter modules, p*GmRD26A*::*GUS* (909 pb) and p*GmRD26B*::*GUS* (435 bp), controlling the expression of the β-glucuronidase (*uidA*) gene. Analysis of GUS activity has demonstrated that p*GmRD26* and p*GmRD26A* induce strong reporter gene expression, as the p*AtRD29* positive control promoter under ABA and PEG treatment.

**Conclusions:**

The full-length promoter p*GmRD26* and the p*GmRD26A* module provides an improved *uidA* transcription capacity when compared with the other promoter module, especially in response to polyethylene glycol and drought treatments. These data indicate that p*GmRD26A* may become a promising biotechnological asset with potential use in the development of modified drought-tolerant plants or other plants designed for stress responses.

## Background

Drought is one of the most limiting and severe abiotic stresses for field crops because it causes significant losses in plants production on a global scale [1, 2]. Under drought conditions, plants trigger many physiological, biochemical, and molecular responses. The sign of abiotic stress is perceived by cellular receptors and secondary messengers, culminating in the gene expression reprogramming to improve plant tolerance, adaptation, and survival. In signal transduction cascade, transcription factors (TFs) emerge as one of the most important messengers in plant adaptation, because they are capable of modifying specific gene expression, encompassing different physiological changes [3, 4]. For example, abiotic factors such as drought, salinity, and heat (high evaporation) alter the osmotic balance in plants’ cell, inducing the biosynthesis of abscisic acid (ABA), a vital phytohormone involved in the expression of drought-related genes [5, 6].

Several TFs are involved in water stress tolerance, including ABA-responsive element (ABRE), nitrogen assimilation control (NAC), dehydration-responsive element binding (DREB), basic leucine zipper (bZIP), myeloblastosis (MYB) and myelocytomatosis (MYC) proteins. All of these TFs are mediators of the classic ABA-dependent or ABA-independent signaling pathways [7–11]. These transcription factors bind preferentially to the dehydration-responsive element (DRE) core sequence (A/GCCGAC) of gene-responsive promoters and regulate several stress-induced genes [12]. The DRE sequence is present in the *A. thaliana RD29A* (*AtRD29*) promoter region and is used extensively to drive expression in a stress-inducible manner in different plants, such as tobacco [13], potato [14], and soybean [15].

The expression of *DREB1A* under the control of the *AtRD29* promoter in *A. thaliana* increased the survival rate of plants stressed with freezing, drought, high salinity, and high temperature [16]. Similar results were also observed in tobacco plants [13]. In both cases, the use of inducible promoter *AtRD29* displays a higher gene expression than the Cauliflower mosaic virus 35S (CaMV35S) constitutive promoter, as it reduces the pleiotropic effects on growth due to the overexpression of *DREB1A* [13, 16]. The expression of genes of interest under the control of the *AtRD29* promoter has been widely used to regulate drought tolerance-associated genes in different plant species [16–19]. However, compared with *AtRD29*, some specific inducible promoters can achieve higher levels of expression. An example is the promoter of the *Coffea arabica CaHB1*2 gene [20]. For this reason, the identification, isolation, and characterization of new specific promoters inducible by abiotic stress have been crucial to ensure the successful application of gene modification and, consequently, the development of new cultivars resistant to water deprivation stress.

In this study, we have investigated the gene expression profile of *GmRD26* in soybean (*Glycine max*), homologous to the *A. thaliana AtRD26* gene (ANAC072). *GmRD26* is highly induced by ABA, PEG, and drought. According to our gene expression analysis, it displays a similar expression profile in comparison with *AtRD26* and *GmNAC085*, a soybean *GmRD26* gene-paralogue already characterized [11, 21]. These genes belong to SNAC-A subfamily (ATAF), as well as *GmRD29*, the *AtRD29* orthologue, extensively used as a model of drought-inducible genes. Previous studies of the GENOSOJA project have demonstrated that *GmRD29* was not differentially expressed during severe water deprivation as *GmRD26* [22], selected as the focus for this study.

Many *SNAC-A* genes are involved with different abiotic stress responses and senescence progression. In soybean, 44% of NAC genes are differentially expressed (DE) during age triggered senescence, being 90% of genes from SNAC-A subfamily [21]. In *A. thaliana*, all SNAC-A gene members - *ANAC055* (AT3G15500), *ANAC019* (AT1G52890), *ANAC072/RD26* (AT4G27410), *ANAC002/ATAF1* (AT1G01720), *ANAC081/ATAF2* (AT5G08790), *ANAC102* (AT5G63790), and *ANAC032* (AT1G77450) - are induced by age triggered leaf senescence [21, 23]. *At*RD26 acts as a transcriptional activator in ABA-mediated dehydration response, positively regulating NYE1, which triggers chlorophyll degradation [24]. The *GmRD26* paralogue in soybean (*GmNAC085*) is also a positive regulator of leaf senescence, displaying high expression during age triggered senescence and classic senescence symptoms when transiently expressed in *Nicotiana benthamiana* [21].

We subsequently isolated and characterized the *GmRD26* promoter (p*GmRD26*). The transcriptional activity of p*GmRD26* and its modules were evaluated in transgenic *A. thaliana* plants under the stress conditions with abscisic acid (ABA), polyethylene glycol (PEG) and drought (air dry) to evaluate the activities of the different regions of the p*GmRD26*.

## Results

### Soybean *RD26* gene expression profile in distinct soybean lines under different stress conditions

**Fig. 1:**
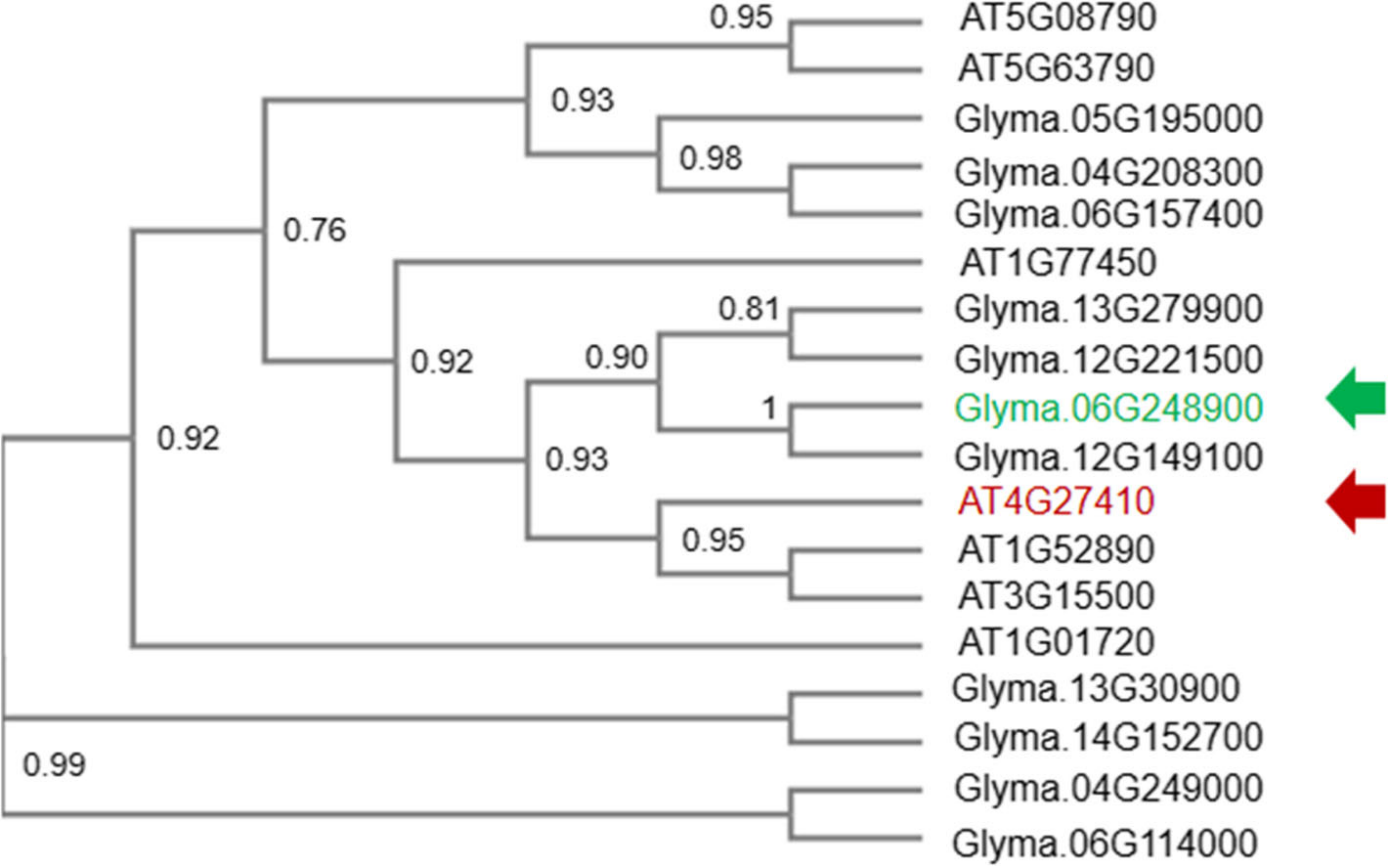
Phylogenetic reconstruction of ATAF soybean genes, members of the NAC transcription factor subfamily. The deduced amino acid sequences of soybean and Arabidopsis were used to perform a multiple alignment using BLASTP and ClustalW2. The phylogenetic tree was constructed using MEGA4.0 software via the neighbour-joining method with a consensus of 10.000 bootstraps. The red arrow indicates the orthologue *A. thaliana* reference gene (AT4G27410), and the green arrow indicates *GmRD26* (Glyma.06G248900)

To identify and characterize the orthologue gene of *A. thaliana AtRD26* (ANAC072) in soybean, an in-silico approach was applied. The *AtRD26* (AT4G27410) sequence was accessed and compared against the Williams 82 soybean reference genome. A putative *RD26* orthologue (*GmNAC043* – Glyma.06G248900) was identified by neighbour-joining analysis, which revealed that at least four genes (Glyma.13G279900, Glyma.12G221500, Glyma.06G248900, and Glyma.12G149100), closely related to *AtRD26*, are present in the soybean genome (Fig. 1). A comparative amino acid deduced sequence analysis of candidate genes was performed, and *GmNAC043*, called *GmRD26,* (Glyma.06G248900) displayed a relatively high amino acid similarity with *AtRD26*.

**Fig. 2:**
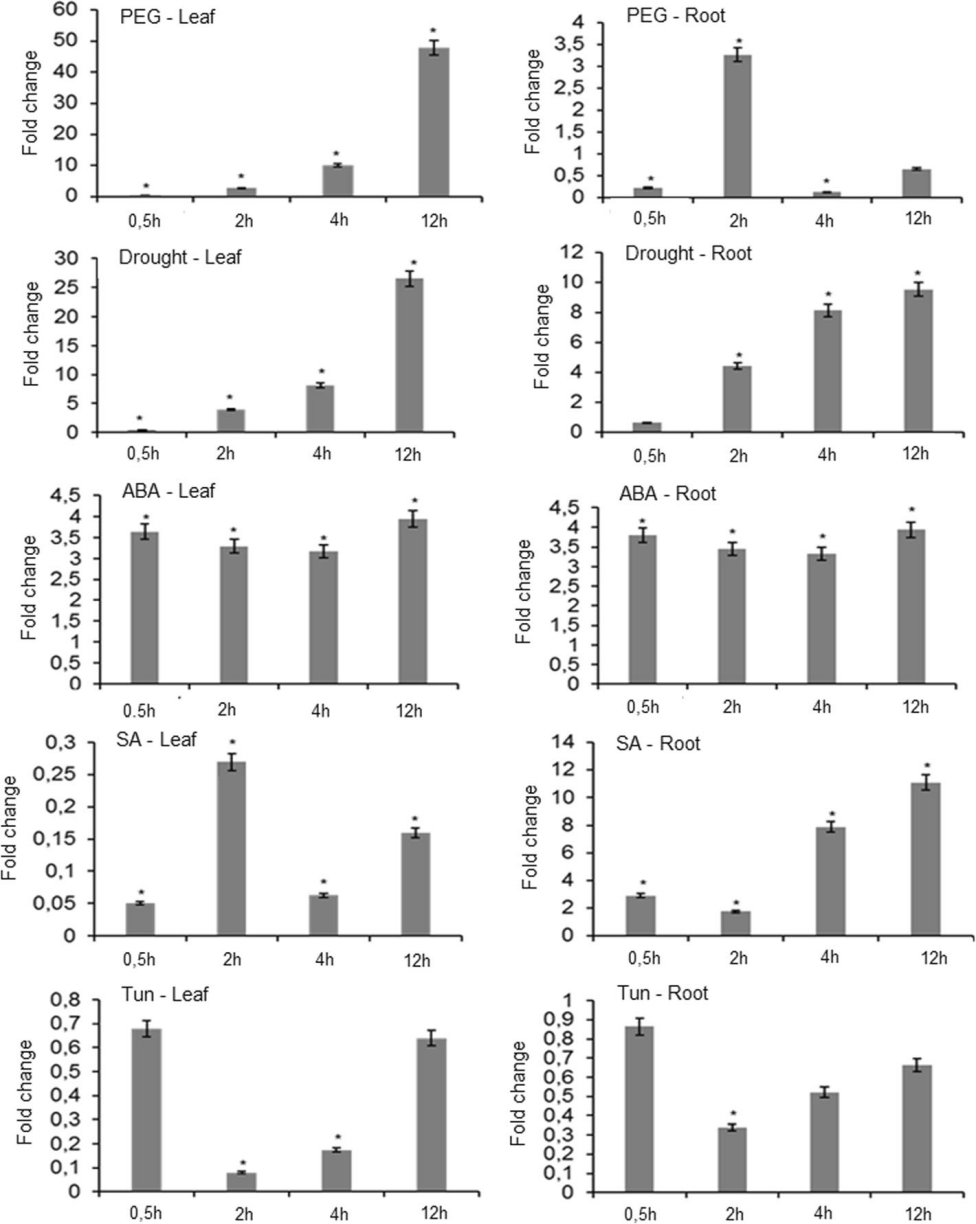
*GmRD26* expression profile in soybean (Williams 82) under multiple stresses. To determine the gene expression profile of the *GmRD26* gene, the soybean seedlings were submitted to different stress conditions (ABA, PEG, SA, Tun and drought), and the gene expression in leaves and roots was analyzed by qRT-PCR. The fold change values were calculated in relation to untreated plants (0 h), considering the relative expression in these plants as 1. *CYP2* and *ELF1A* were used as endogenous controls for normalization. The relative gene expression was calculated by the 2^-ΔΔCt^ method in biological triplicates (*n* = 3). The bars represent standard errors and the asterisks (*) indicate statistical significance determined by the Student’s *t*-test (*p* ≤ 0.05)

To evaluate whether the *GmRD26* soybean transcription factor is induced during water stress, its expression pattern was analyzed in cDNA subtractive libraries related to dissection experiments available in the GENOSOJA LGE (Genomics and Expression Laboratory: GENOSOJA Project) database and from these analyses, the presence of *GmRD26* was confirmed. To evaluate the *GmRD26* expression profile and its relation to multiple stress responses in soybean, the transcript levels were analyzed in the leaves and roots of Williams 82 soybean seedlings by qRT-PCR. The expression pattern was also evaluated for *GmNAC085*, a paralogue of *GmRD26* gene whose stress induction profile is reported previously [21]. As expected, *GmRD26* is highly expressed under the use of PEG (10% m/v) in leaves but is also induced by ABA (150 mM) and drought in leaves and roots (Fig. 2). This gene expression profile is similar to the *GmNAC085* expression (Additional file 1: Figure S1). In addition, both related genes are repressed by tunicamycin (Tun) in leaves and roots, showing an expressive induction by salicylic acid (SA) (5 mM) treatment in roots (Fig. 2; Additional file 1: Figure S1).

**Fig. 3:**
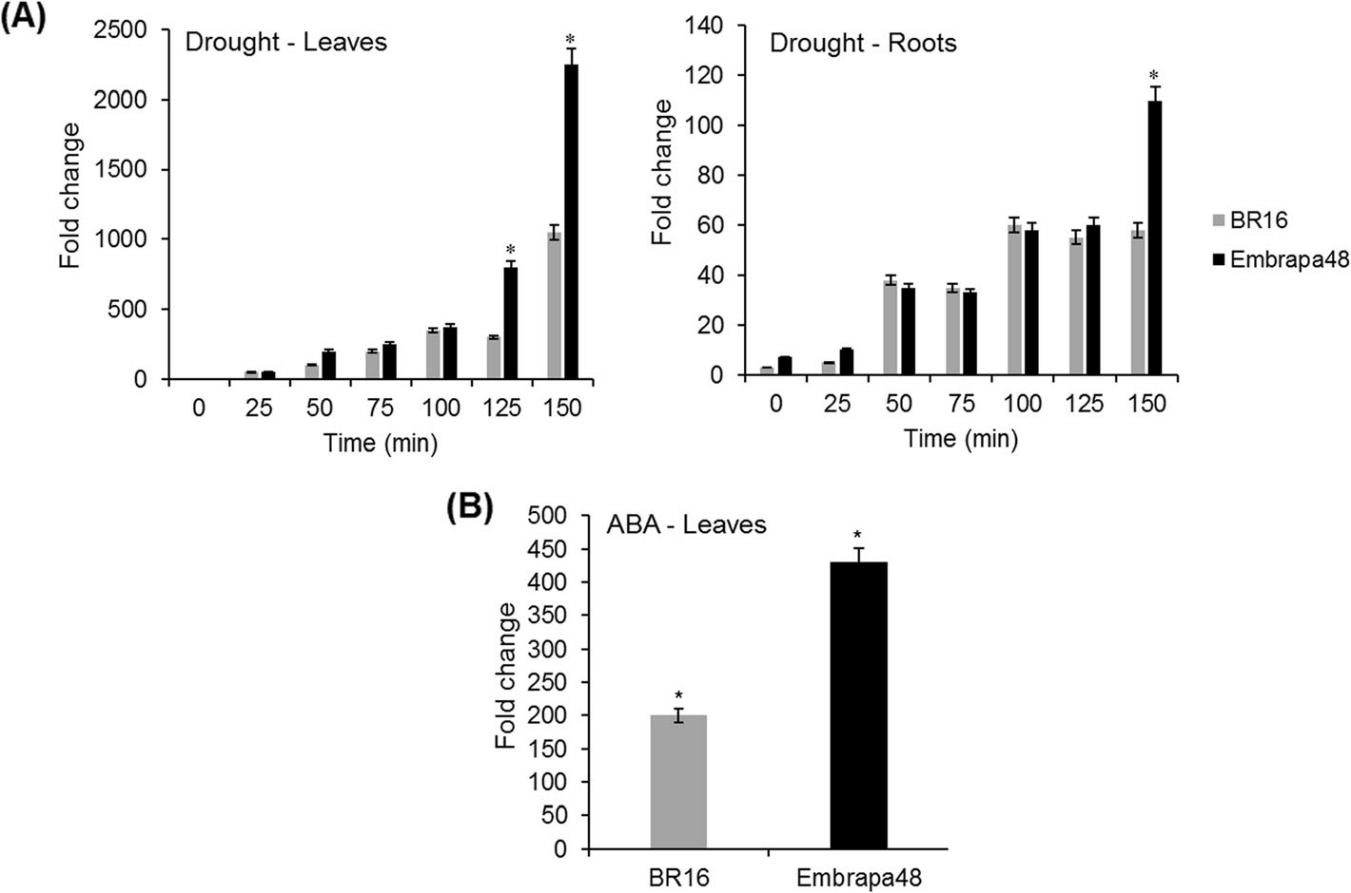
*GmRD26* expression profile in two soybean cultivars, the drought-susceptible BR16, and the drought-tolerant EMBRAPA48. **a** Expression profile of the *GmRD26* gene under drought conditions, the gene expression pattern was determined at 25, 50, 75, 100, 125, and 150 min after water deprivation. **b** Expression profile of the *GmRD26* soybean gene in the leaves of contrasting genotypes BR16 and EMBRAPA48 after 6 h of exogenous ABA stimuli. *CYP2* and *ELF1A* were used as endogenous controls for normalization. The relative gene expression was calculated by the 2^-ΔΔCt^ method in biological triplicates (n = 3). The bars indicate the standard errors and the asterisks (*) indicate statistical significance determined by Student’s *t*-test (*p* ≤ 0.05)

The expression profile of *GmRD26* was also determined in two contrasting soybean genotypes in response to drought tolerance under simulated drought stress (Fig. 3) and ABA exogenous stimuli (Fig. 3a). It is expected that positive regulators of drought perception, signal transduction, and drought avoidance-associated genes are expressed higher in tolerant lineages than in susceptible lineages, as shown in the gene expression analysis results. In addition, the gene expression-folding is extensively high in BR16 and Embrapa48 cultivars when compared with the expression in Williams 82.

*GmRD26* was differentially expressed in both leaves and roots of contrasting cultivars, and the tissues display a similar induction pattern as observed in Williams 82 under PEG (10% m/v) stress (Figs. 2 and 3). In the roots, the gene expression was considerably lower than in the leaves. The difference between the cultivars is the gene expression levels: the susceptible cultivar BR16 had a significantly lower *GmRD26* transcript accumulation in comparison with the tolerant cultivar Embrapa48 at all times of stress progression (Fig. 3). The gene expression significantly increased beginning at 125 min, showing that the *GmRD26* gene is strongly induced under severe stress conditions. The ABA response was also analyzed. As observed in *Arabidopsis*, the results revealed that *GmRD26* is also up-regulated by ABA in both soybean cultivars and the mRNA levels are significantly higher in tolerant cultivar Embrapa48, as observed in drought treatment (Fig. 3a and b).

### Analysis of water deficit-responsive *cis*-elements frequency

**Fig. 4:**
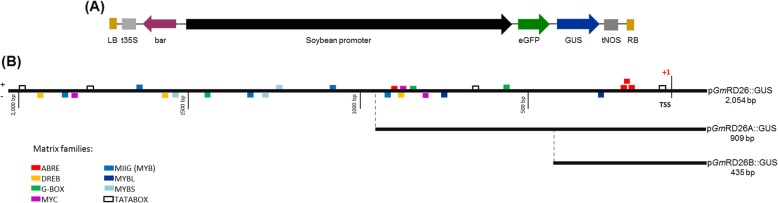
Schematic representation of the *GmRD26* promoter regions controlling the expression of the *GUS* reporter gene. **a** Schematic drawing of the soybean promoter expression cassette in the p*C1149*::*GUS* expression vector. **b** Diagram of the main *cis-*acting elements in the full-length p*GmRD26* (2.054 bp) promoter and the modular promoters p*GmRD26A* (909 bp) and p*GmRD26B* (435 bp). The families of *cis*-elements were identified using the Genomatix databases (*p*-value ≤0.05) and are represented by coloured boxes

**Fig. 5:**
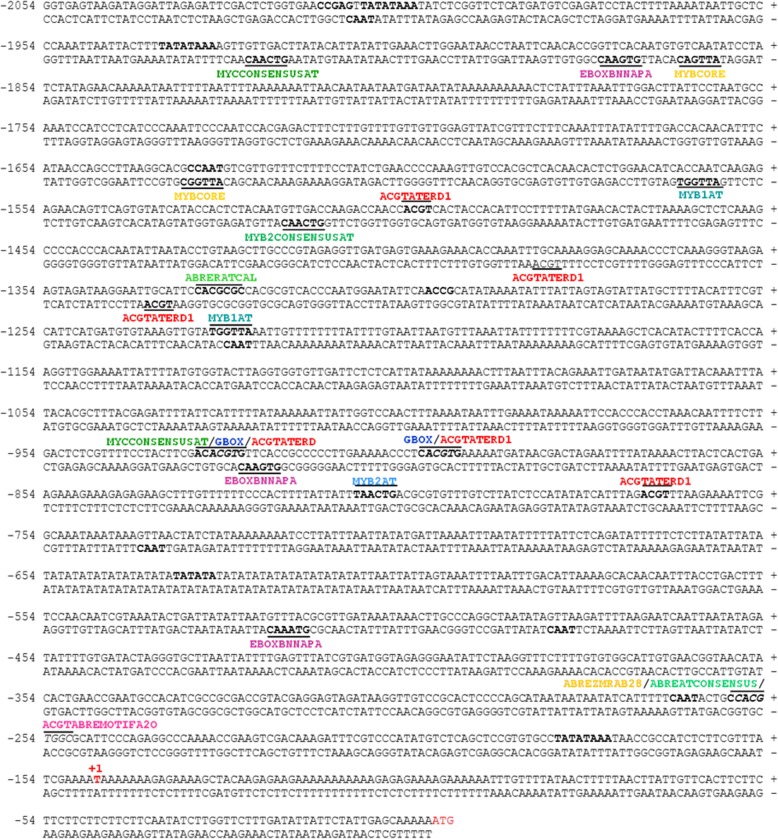
Physical map of the *GmRD26* promoter. The transcription start site is highlighted in red and is designated as + 1. The TATA-box is highlighted in bold. The numbers on the left side indicate the distance from the transcription start site. The sequences were analyzed by Genomatix databases (p-value ≤0.05). The putative *cis-*elements provided in p*GmRD26* are indicated by a bar and their names. Sense acting motifs (5′– 3′) are indicated by a superior bar, while antisense acting motifs (3′– 5′) are indicated by an inferior bar. All the stress-responsive motives are represented by different colours

To investigate the transcriptional activity of the *GmRD26* soybean promoter under different stress conditions, the full-length promoter sequence (2.054 bp) was analyzed using PLACE and Genomatix for *cis-*regulatory element mapping. The promoter sequence analysis revealed some conserved TATA- and CAAT-box regions that are essential for transcription initiation complex assembly and gene transcription in eukaryotes. Potential *cis-*regulatory element families such as the ABRE, DREB, G-box, MYC and MYB families, which can respond to many environmental signals, abiotic stresses and phytohormones were also found in the p*GmRD26* sequence (Fig. 4 and Table 1). The families’ distribution in each promoter module used for *A. thaliana* genetic transformation is represented in Fig. 4b. Our analysis also revealed some specific drought-responsive *cis*-elements, MYB2AT and ACGTATERD1, as well as ABA-responsive ones, ABRERATCAL, ABREATCONSENSUS, DPBFCOREDCDC3, and EBOXBNNAPA Moreover, in the p*GmRD26* sequences, some doubly responsive elements, MYB2CONSENSUSAT, ABREZMRAB28, MYBCORE, and G-box, have been identified that respond to both drought and ABA. (Fig. 5 and Table 1). The most frequent *cis*-elements identified in the modular p*GmRD26A* (909 bp) and p*GmRD26B* (435 bp) were DPBFCOREDCDC3, ABRERATCAL, and ABREATCONSENSUS, required in ABA-signaling and MYCCONSENSUSAT, ACGTATERD1 and MYBCORE, involved in dehydration-responses (Table 1). High-salinity responsive *cis*-elements are also present. These stress-associated *cis*-elements were also found in *AtRD29* promoter, and it was observed that *GmRD26* promoter has nine of thirteen dehydration and ABA-responsive *cis*-elements, as found in p*AtRD29* promoter (Additional file 2: Table S1).

**Table 1:** *Cis*-regulatory elements related to drought in the p*GmRD26* soybean promoter

*Cis*-regulatory element	Core sequence	Description	References
ACGTATERD1	ACGT	Dehydration	[25]
MYCCONSENSUSAT	CANNTG	Dehydration, ABA and Cold	[26, 27]
ACGTABREMOTIFA2OSEM	ACGTGKC	Dehydration and ABA	[28]
DRE2COREZMRAB17	ACCGAC	Dehydration and ABA	[29]
MYB2CONSENSUSAT	YAACKG	Dehydration and ABA	[30]
ABREZMRAB28	CCACGTGG	ABA-responsive	[31]
ABREATCONSENSUS	YACGTGGC	ABA-responsive	[32, 33]
MYBCORE	CNGTTR	Dehydration and ABA	[34, 35]
MYB1AT	WAACCA	Dehydration and ABA	[30]
MYB2AT	TAACTG	Dehydration	[34]
G-box	CACGTG	Dehydration, high salinity, ABA	[36]
EBOXBNNAPA	CANNTG	ABA-responsive	[26, 37]
DPBFCOREDCDC3	ACACNNG	ABA-responsive	[38, 39]
ABRERATCAL	MACGYGB	ABA-responsive	[40]

K = G/T; R = G/A; W = A/T; N = A/C/G/T; Y = *T C*

### GUS activity and expression in transgenic *A. thaliana* lineages under p*GmRD26* control during different stress treatments

**Fig. 6:**
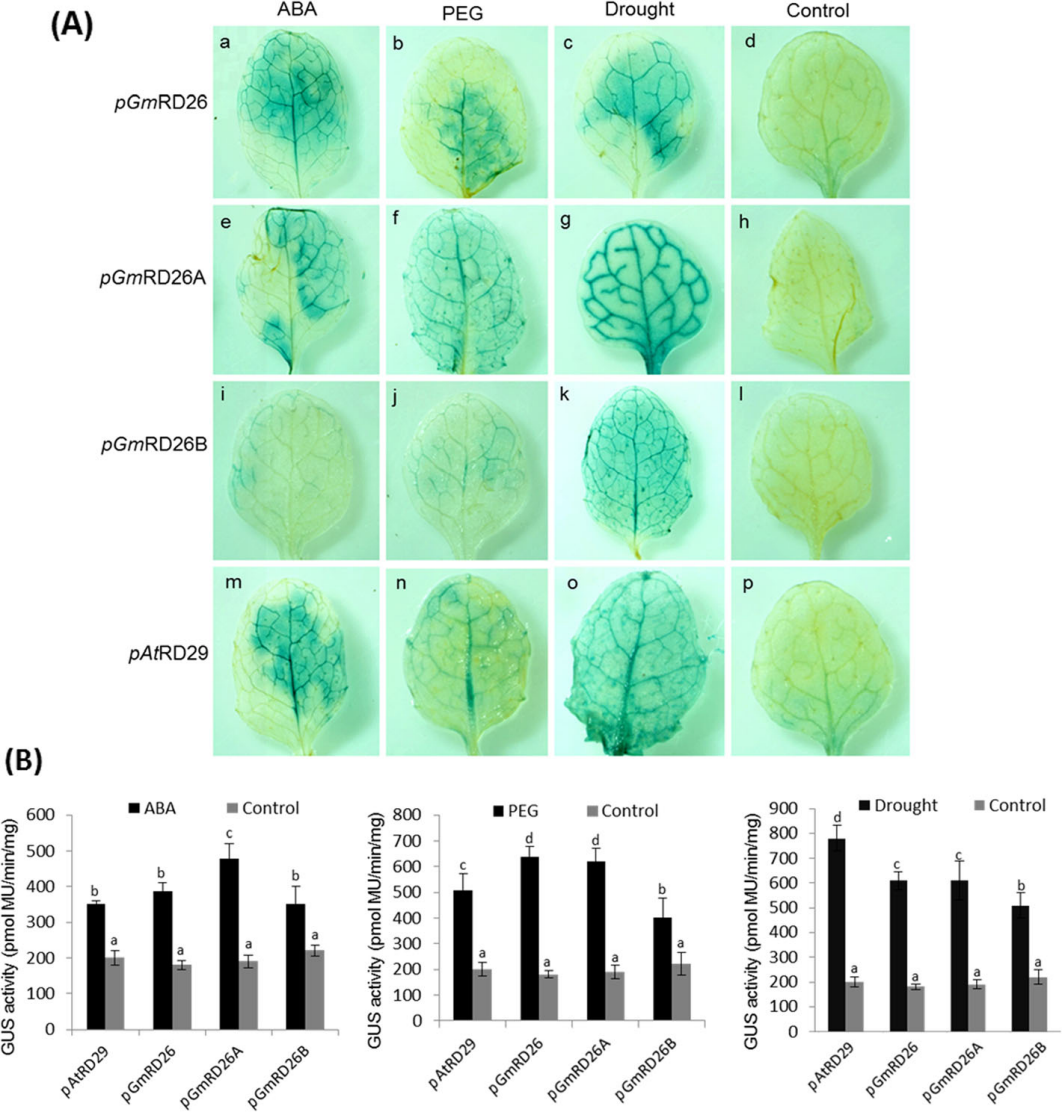
Histochemical and quantitative fluorimetric analysis of different *GmRD26* promoter deletion constructs in transgenic *A. thaliana* plants. The stress treatments for GUS activity analysis was performed on 4-week-old *A. thaliana* plants under 12 h treatments with ABA, PEG, or drought. **(A)** Histochemical localization of GUS activity in transgenic *A. thaliana* plants harboring promoter-*GUS* constructs. **(B)** The quantitative fluorimetric assay for GUS activity was carried out in three replicates. The soybean promoter and its modules were compared with the p*AtRD29* and wild type plants. Control samples consisted of untreated plants. Bars indicate standard error and different lowercase letters indicate significant differences at *P* < 0.05 on Tukey’s Test. The data shown are representative of three independent lines (n = 3)

Homozygous T_2_ *A. thaliana* lineages carrying the full-length p*GmRD26*::*GUS* and the promoter modules p*GmRD26A*::*GUS* and p*GmRD26B*::*GUS* were used to analyse promoter induction under drought stress through GUS activity. The GUS histochemical assay was performed after 12 h of treatment with ABA and PEG in transgenic lineages and the controls p*AtRD29*::*GUS* (positive control) and non-treated plants (negative control). Plants carrying the p*GmRD26*::*GUS* and p*GmRD26A*::*GUS* displayed intense GUS activity in their foliar vascular tissue after ABA treatment, as well the positive control p*AtRD29* (Fig. 6A- a, e, m). In contrast, the p*GmRD26B*::*GUS* is not strongly inducible by ABA, according to its GUS activity (Fig. 6A - i), although ABRE elements are abundantly distributed in this promoter module. Under PEG treatment, the GUS activity pattern was similar (Fig. 6A - b, f, j, n), with a discrete decline in activity in the p*GmRD26*::*GUS* plants when compared with ABA and drought (air dry) treatment (Fig. 6A - b). In the p*GmRD26A*::*GUS* construct, a strong GUS-derived staining was observed in almost all the leaves surfaces in PEG treatment (Fig. 6A - f). Under the drought treatment, GUS activity was strongly detected in all analyzed leaves, mainly in the modular constructs p*GmRD26A*::*GUS*, p*GmRD26B*::*GUS* (Fig. 6A - g and k). The basal expression in the control plants (without stress conditions) was low but detectable (Fig. 6A - d, h, l, and p). In our study, *pGmRD26A* displays activity in all treatments, but this activity is higher under desiccation conditions (Fig. 6A - g), reinforcing the role of RD26 in desiccation-triggered protective mechanisms in plants.

**Fig. 7:**
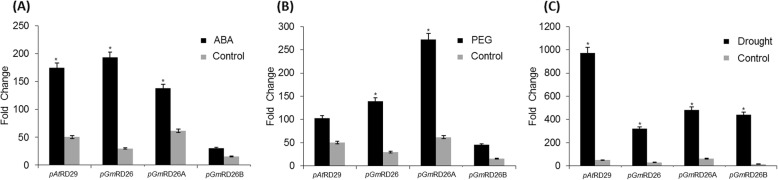
Transcriptional *GUS* activity in transgenic *A. thaliana* under 12 h of ABA (**a**), PEG (**b**) or drought (**c**) treatments. Levels of *uidA* mRNA of non-treated (control) and treated transgenic plants were measured using real-time PCR. The expression levels were normalized using *ACT2* and *GAPDH* as endogenous controls. The relative gene expression was calculated using the 2^-ΔΔCt^ method. The bars represent standard error and the asterisks (*) indicate statistical significance determined by Student’s *t*-test (*p* ≤ 0.05). The data shown are representative of three independent lines (n = 3)

To confirm the induction profile of p*GmRD26* revealed by histochemical assays, GUS activity was also monitored in transgenic lineage plants. Under ABA treatment, full-length p*GmRD26* encompassed the same results when compared to the p*AtRD29* positive control, and the module p*GmRD26A* displays the higher GUS activity (Fig. 6B). These results contrast with the PEG treatment, in which the full-length promoter and the p*GmRD26A* module exhibit higher activity when compared with the positive control, p*AtRD29*, and the p*GmRD26B* (Fig. 6B). When GUS activity was analyzed under drought treatment, p*GmRD26A* shows the same activity of the full-length promoter, higher than the smaller module p*GmRD26B*, but lower than the positive control. The activity of the p*GmRD26A* module was higher than the other fragments, and the p*AtRD29* control under ABA and PEG treatments (Fig. 6B). In addition, p*GmRD26A* transgenic lines display high levels of *uidA* mRNA after PEG treatment, while p*GmRD26* lines display high levels of *uidA* transcripts under ABA treatment. In the drought treatment, p*AtRD29* control lines presented higher expression level than the p*GmRD26* promoter and its modules. However, when we analyzed the differences between the three fragments after drought treatment, p*GmRD26A* showed higher expression levels compared to p*GmRD26* and p*GmRD26B* (Fig. 7).

The results of qPCR demonstrate that transcriptional GUS activity, driven by the promoters p*GmRD26* and p*GmRD26A* was similar but not the same during ABA treatment. According to our data, p*GmRD26* display a similar expression when compared with p*AtRD29* (positive control) and higher mRNA accumulation when compared with p*GmRD26A* module (Fig. 7a). This data is compatible with the histochemical assay. Under PEG treatment, the module p*GmRD26A* displayed a higher transcriptional activity, followed by the full-length p*GmRD26* and the positive control p*AtRD29* (Fig. 7b). The module p*GmRD26B* continued to displaying lower *GUS* transcriptional activity. As expected, under drought condition, p*AtRD29* displayed higher *GUS* expression, followed by considerable *GUS* expression driven by modules p*GmRD26A* and p*GmRD26B* and the full-length promoter p*GmRD26* (Fig. 7c).

## Discussion

In this study, we confirmed that the *GmRD26* gene is induced under different simulated drought conditions. In view of the potential of this gene as a target for the development of strategies for the genetic engineering of resistant plants, we decided to isolate and characterize the *GmRD26* promoter region. Our results show that p*GmRD26* and its modules activated the reporter gene *uidA* under different water deprivation stress conditions. These results are consistent with the characteristics of the *cis*-regulatory elements identified by in silico analyses of p*GmRD26* sequence. In Arabidopsis, *At*RD26 is an important member of ABA-dependent drought tolerance, and its overexpression is associated with a drought-tolerant phenotype [41].

The *GmRD26* gene, as well as its *A. thaliana* orthologue (*AtRD26*/ANAC072), belongs to the subfamily SNAC-A, whose members have some correlation of functional conservation with the *ATAF1* gene (AT1G01720), which has been shown to be a regulator of ABA biosynthesis and responsive to water stress [42–46]. Analysis of *GmRD26* expression in two contrasting drought-tolerant soybean cultivars, BR16 and Embrapa48, and in the Williams 82 (soybean reference genome) demonstrated that the expression profile of the *GmRD26* gene is compatible with phylogenetic and molecular characteristics already described for orthologue genes, reinforced by *GmNAC085* expression profile, a phylogenetically close-related gene in soybean [42, 47]. Our gene expression analysis reveals that the induction fold of *GmRD26* is not the same along the three analyzed cultivars, displaying an unexpectedly high expression level in BR16 and Embrapa48. The different genetic background of these cultivars should proportion this difference, once the BR16 and Embrapa48 are commercial cultivars, obtained by genetic breeding programs. In our study, *GmRD26* was responsive to osmotic stress during PEG treatment, desiccation and exogenous ABA stimulation in both leaves and roots, while the tolerant soybean variety displays higher gene expression level than susceptible variety. The leaves exhibited a more significant folding variation, suggesting that the physiological mechanism triggered by *GmRD26*, mainly related to ABA-dependent responses, is more effective in the leaves than in the roots. In general, genes involved in ABA-mediated stress responses are involved in leaf morphophysiological changes, including stomatal closure, leaf area adjustment, photosynthesis, transpiration index, and osmolyte accumulation [48, 49]. In Arabidopsis, it was already demonstrated that *GmNAC085* overexpression confers drought tolerance, improving the plant physiological performance during water deprivation stress. The transgenic lineages display a more robust antioxidative response under stress and many readouts genes, involved in ABA-dependent signalling, are up-regulated [11]. These results, associated with the determined *GmRD26* and *GmNAC085* gene expression profile, may justify the drought inducibility of SNAC-A genes during abiotic stress and confirm their potential to drive expression of genes involved with plant adaptability.

Compared with p*AtRD29*, a previously characterized drought-associated promoter, the promoter p*GmRD26* was also enriched in stress-related *cis-*elements, responsive to salinity, dehydration, ABA and temperature. These results can be directly related to the excellent performance of the soybean promoter under ABA and PEG treatments. During drought treatment, the promoter displays some reasonable activity but is not capable of being compared with p*AtRD29*. This broad responsive promoter activity can be applied in soybean molecular breeding programs. In ABA-dependent pathways, ABREs (ABA-responsive elements) are the main phytohormone-responsive *cis-*element [43]. The occurrence of three ABRE motifs from five total ABA-responsive elements indicates a strong promoter induction under drought conditions, which can trigger increased drought-responsive gene expression by p*GmRD26* during stress. This effect is reinforced by the presence and frequency of the ACGT motif, a characteristic and important *cis-*element in drought-responsive promoters [50]. *Cis*-acting elements of the G-box family, found in several plant genes’ promoters are known to interact with bZIP transcription factors, mediating responses to different stimuli. Studies comparing the patterns and evolution of the G-box family core (ACGT) in *O. sativa*, *S. bicolor*, *A. thaliana* and *G. max* suggest that this is the family with the most conserved elements between species and leads to responses to exogenous stresses, especially water and salt stress [51]. Other stress-responsive elements are also present in p*GmRD26*, such as MYCs/MYBs, which exhibit rapid induction in response to ABA treatment and water stress. These elements are targets of a large TF family in soya. MYC and MYB transcription factors are necessary for the early response to osmotic stress [43, 52].

In this study, we also the activity of the soybean promoter p*GmRD26* and two-promoter modules, p*GmRD26*A and p*GmRD26*B, in transgenic *A. thaliana* plants that were submitted to simulated (ABA and PEG) and real drought stress. The p*GmRD26*, p*GmRD26A*, and p*GmRD26B* promoters were induced by all stress treatment assays, showing greater or similar GUS activity than p*AtRD29* (positive control) under ABA, PEG, and drought treatment. Differences in induction intensity between p*GmRD26* and modules under different types of abiotic stress are probably related to the distribution of specific *cis*-elements in their sequences involved in the control of water stress response [53, 54]. The transcriptional activation of some genes depends not only on the promoters’ *cis*-acting elements and their sequences but also on their position and the presence of enhancers, regulatory sequences and other synergistic *cis*-elements [55, 56]. It is important to highlight that some differences between transcriptional and translational activity are common on promoter’s genes analysis. Our data demonstrate that the induction profile of p*GmRD26* is similar in the tested conditions, showing that the full-length promoter and its modules respond to the same conditions. Also, promoters’ modules enriched in *cis*-acting elements drive more consistent gene expression, reinforcing the idea of a synergistic effect of *cis*-elements in gene promoter sequences.

Similar results were obtained in the characterization of the α-galactosidase soybean promoter (*GlymaGAL*) responsive to water stress; the smallest fragment, p*GAL*-1 kb, showed no significant difference in GUS activity compared to the control and treated samples (PEG and dry). The full-length fragment promoter, p*GAL*-2 kb, however, led to a significant increase in *GUS* expression. This increase in *GUS* expression of p*GAL*-2 kb was associated with a high number of ABRE, MYCATERD1, G-box, and DRE *cis*-elements [57]. Other studies have also reported the importance of distal promoter regions in responses associated with water stress in other species [58, 59].

## Conclusions

In this study, we analyzed the expression profile of *GmRD26* gene, which is expressed under simulated osmotic and drought conditions in soybean. The stressed soybean seedlings display a high *GmRD26* expression under ABA exogenous-stimuli in leaves and roots, as well under PEG and air dry treatment. This gene expression pattern raised the hypothesis of drought-inducible *cis*-elements enriched promoter of *GmRD26*. Our analysis showed that the *GmRD26* promoter region is enriched with essential *cis*-elements associated with drought stress, such as ABRE, DREB, MYB, MYC, and G-BOX. Molecular characterization of p*GmRD26* in *A. thaliana* has demonstrated that the full promoter (p*GmRD26*) and two different promoter-modules (p*GmRD26A* and p*GmRD26B*) are inducible under simulated osmotic and drought stress conditions, confirming the soybean gene expression profile. In addition, our data also revealed that the full-length promoter and the p*GmRD26A* module, with higher *cis*-acting elements incidence compared to the other module, displayed a slightly higher level of expression than p*GmRD26B* and the p*AtRD29*, an *A. thaliana* promoter used as a model to drought inducible gene studies, during ABA and PEG treatment. The complete characterization of p*GmRD26* and its modules suggests that the promoter or the fragment p*GmRD26A* may become a potential biotechnological tool capable of inducing expression of genes of interest under specific conditions, such as drought or other abiotic stresses related with an osmotic imbalance to improve the tolerance associated to physiological performance in genetically modified plants.

## Methods

### Identification of the drought marker gene *GmRD26* in soybean

The *A. thaliana* RD26 (AT4G27410) deduced amino acid sequence (available in TAIR database - https://www.arabidopsis.org/) was used to identify the closely related orthologue gene (*GmRD26*/*Gm*NAC043 - Glyma.06G248900) in the soybean genome (Williams.82 v2.2-available in Phytozome: https://phytozome.jgi.doe.gov) [60]. For sequence comparison, BLASTP (https://blast.ncbi.nlm.nih.gov) was used, and the alignment was confirmed using the online tool ClustalW2 (https://www.ebi.ac.uk/Tools/msa/clustalw2). To determine the phylogenetic relationship between the Arabidopsis and soybean genes, the neighbour-joining clustering method derived from a distance matrix from a Poisson model was used, and the tree was reconstructed using MEGA software [61].

To evaluate whether the selected putative soybean gene *GmRD26* is induced during drought stress, its expression pattern was analyzed from cDNA subtractive libraries related to dissection experiments available in the GENOSOJA database LGE (Genomics and Expression Laboratory: GENOSOJA Project) [22].

### Soybean plant growth conditions and stress treatments

For the *GmRD26* gene expression profile analysis, soybean (Williams 82) seeds were germinated in the soil and grown under greenhouse conditions (12 h of light, 25–35 °C, 70% relative humidity) until the V2-V3 development stage. To simulate multiple stress conditions, the seedlings were first transferred to Hoagland hydroponic solution for 24 h. After acclimation, the soybean roots were immersed in the same solution supplemented with 10% (w/v) PEG 8000 to induce osmotic stress, 5 μg/mL tunicamycin (Tun) to induce endoplasmic reticulum stress, 150 mM ABA and 5 mM salicylic acid (SA) to simulate drought and biotic stress conditions, respectively. For the drought treatment, the plants were removed from the hydroponic solution and placed on plates with cotton. Leaf discs and roots of treated and control (0 h - untreated plants were collected one time) seedlings were collected after 0.5 h, 2 h, 4 h and 12 h of stress treatment and immediately frozen in liquid nitrogen. All treatments were performed at a three-plants pool, and samples were collected in triplicate.

Embrapa48 and BR16 soybean cultivars were used to determine the expression profile during the ABA and drought treatments in drought responses contrasting soybean lineages. The BR16 variety is considered as a model of drought sensitivity, while Embrapa48 is considered as drought-tolerant cultivar. The seeds were germinated in watered germination test paper and then transferred to a hydroponic box system filled with Hoagland solution. The seedlings in stages V3-V4 were grown under the same conditions as the Williams 82 seedlings. The drought stress was generated by removing the plants from the hydroponic solution and placing them in empty boxes for different water deprivation periods: 0 min (T0 - control), 25 min (T25), 50 min (T50), 75 min (T75), 100 min (T100), 125 min (T125) and 150 min (T150). Roots and leaf disc samples from three plants of each cultivar were collected during the exposure to water and were immediately frozen in liquid nitrogen for RNA extraction and gene expression analysis.

The contrasting soybean cultivars were also submitted to exogenous ABA treatment. Plants germinated and grown under the same conditions were sprayed with water (control) or ABA solution (300 ppm). Three biological replicates were used, consisting of three plants per treatment. After 6 h, leaf discs were collected and immediately frozen in liquid nitrogen for RNA extraction.

### RNA extraction, cDNA synthesis and *GmRD26* gene expression analysis

The total RNA of soybean leaf and roots was extracted according to the TRIzol® manual (Invitrogen, USA). RNA quantification was performed using a NanoDrop™ Spectrophotometer ND-1000 (Thermo Scientific, USA) and the RNA integrity was assessed by 1% agarose gel electrophoresis. A total of 2 μg of RNA was used for cDNA synthesis with MMLV reverse transcriptase protocol (Invitrogen, USA).

The gene expression profile was determined by qPCR. The analysis was performed using an ABI 7500 Fast instrument, SYBR Green (Invitrogen, USA) reagent, specific primers (Additional file 3: Table S2) and three independent cDNA pools. All the analyses were performed using three biological and two technical replicates, originated from a five soybean plants pool. The reaction was performed as follow: 2 min at 50 °C, 10 min at 95 °C, and 40 cycles of 94 °C for 15 s and 60 °C for 1 min. The *CYP* and *ELF* soybean genes [62] were used as endogenous controls for expression normalization and relative gene expression calculated by the 2^-ΔΔCt^ method. The endogenous gene stability was determined by G-norm algorithm (https://genorm.cmgg.be/), from Q-base package, and the M-value is 0.89 and 0.91 for *CYP* and *ELF*, respectively. The *GmRD26* orthologue gene, *GmNAC085*, was used as a comparative control in Williams 82 for gene profile determination.

### Analysis of p*GmRD26* soybean *cis*-acting elements

The p*GmRD26* promoter sequence (2.054 bp) was obtained from the soybean genome available in the Phytozome database (https://phytozome.jgi.doe.gov) [60]. The *cis*-acting elements responsive to drought-, salinity-, osmotic- and ABA-induced stress were identified, analyzed, and mapped using the Genomatix (https://www.genomatix.de/online_help/matinspector/matinspector). For this study, we considered only the *cis*-elements statistically significant, with a *p*-value ≤ 0.05 [63, 64].

### Construction of p*GmRD26* plasmids

The full-length *GmRD26* soybean promoter region was considered as the 2.054 bp gene-promoter, and A and B promoter-modules contain 909 bp and 435 bp, respectively, considering the distribution of the drought-responsive *cis-*acting elements*.* The sequences were transcriptionally fused in frame to the *GUS* gene in a binary expression pC1407 vector backbone, synthesized by Epoch Biolabs (Sugar Land, TX, USA). The generated recombinant plasmids were called p*GmRD26*::*GUS* (2.054 bp), p*GmRD26A*::*GUS* (909 bp) and p*GmRD26B*::*GUS* (435 bp). The plasmids carry out the translational *GUS-GFP* fusion and BaR plant selection marker gene. The *AtRD29A* (*AtRD29*) promoter gene sequence [65] was cloned into the same plasmid as a positive control of drought-inducible promoters.

### Transgenic *A. thaliana* plants

The recombinant plasmids were introduced into *Agrobacterium tumefaciens* GV3101 strain, which was then transferred to *A. thaliana* ecotype Columbia (Col-0) by floral dip method [66]. Transgenic plants with a T-DNA insertion were identified by glufosinate-ammonium selection and confirmed by PCR. Three homozygous independent lines were obtained for each construction and T_2_ plants expressing *GUS*-GFP used in abiotic stress treatments and promoter characterization.

### Drought, PEG and ABA treatment of *A. thaliana* transgenic lineages

The *A. thaliana* seeds were germinated in the soil and grown under growth chamber-controlled conditions (12 h photoperiod, 21 °C temperature and 70% relative humidity). After 10 days, the seedlings were sprayed three times at intervals of 5 days with glufosinate-ammonium (100 mg/L) for positive transgenic plant selection. Four weeks old transgenic plants were carefully removed of soil moisture, and their roots were immersed in Hoagland hydroponic solution supplemented with 5% (w/v), PEG (MW 8.000) and 50 μM ABA solution to simulate drought conditions. For the drought treatment, the plants were removed from the hydroponic solution and placed on open plates. The non-stressed controls consisted of plants that were kept in Hoagland hydroponic solution. Two leaves of three plants for each full-length or modular promoter were collected after 12 h of treatment and immediately frozen in liquid nitrogen and stored at − 80 °C for further extraction of RNA.

### Histochemical GUS assays

To detect GUS activity in transgenic *A. thaliana* lineages, fresh leaves were incubated for 12-16 h at 37 °C in 5-bromo-4-chloro-3-indolyl-β-D-glucuronic acid (X-Gluc) solution [67]. After X-Gluc incubation, the leaves were washed with water, and the chlorophyll was removed with ethanol (70% v/v) for approximately 10 h. The leaves were washed and then observed under Leica Wild Heerbrugg M3Z Stereozoom Microscope (Leica, Wetzlar, Germany). For each construct, leaves were collected from at least three different transgenic plants lineages.

### Fluorimetric GUS assay

The *A. thaliana* transgenic plants were grouped into three-plant pools and subjected to stress treatments (PEG, ABA, and drought) as previously described. For plants pool protein extraction, extraction buffer with 100 mM NaH_2_PO_4_, 0.01% SDS, 10 mM EDTA, 0.1% sodium lauryl sarcosine, 0.1% Triton X-100 and 1 mM DTT was used. Protein extraction was performed with frozen tissue powder (~ 100 mg), and samples were manipulated on ice. The total soluble proteins were quantified by the Bradford method [68] and used for a fluorimetric assay. The fluorimetric GUS assay was performed in a 500 μL reaction consisting of 400 μL of protein extract and 100 μL of 10 mM 4-methylumbelliferyl β-D-glucuronide (MUG; Sigma, USA). The reaction was incubated for 1 h at 37 °C. At the start point, a 50 μL reaction aliquot was removed and added to 450 μL of 0.2 M Na_2_CO_3_ stop buffer. The fluorescence of 4-methylumbelliferone (4-MU) was monitored using a Versa Fluor Fluorometer (BioRad) with excitation at 365 nm and emission at 455 nm. Each sample was analyzed in triplicate, and values were calculated according to a reference range of MU. GUS activity was expressed in nanomoles of MU produced per minute per microgram of soluble protein.

All GUS fluorimetric assays were repeated at least three times. The results were expressed as the mean of independent experiments with the respective standard error. Different lowercase letters above the bars indicate significant differences at *p* < 0.05.

### *GUS* gene expression analysis

The *GUS* gene expression was analyzed in transgenic *Arabidopsis* plants expressing p*GmRD26*::*GUS*, p*GmRD26A*::*GUS*, p*GmRD26B*::*GUS* and p*AtRD29*::*GUS*. The gene expression level was monitored by qRT-PCR using three biological and two technical replicates, as previously described for soybean genes; the expression levels were normalized using *ACT2* (AT3G18780 [69]) and *GAPDH* (AT1G13340 [70]) as endogenous controls. The endogenous gene stability was determined by G-norm algorithm, from Q-base package, and the M-value is 0.86 and 0.79 for *ACT2* and *GAPDH*, respectively. The primers used are described in Additional file 3: Table S2.

## Supplementary information

**Additional file 1: Figure S1**. *GmNAC085* expression profile in soybean (Williams 82) under multiple stresses. To determine the gene expression profile of the *GmNAC085* gene, the soybean seedlings were submitted to different stress conditions (ABA, PEG, AS, Tun, and drought), and the gene expression were analyzed in leaves and roots by qRT-PCR. The fold change values were calculated in comparison of plants treated with untreated plants (0 h). *CYP2* and *ELF1A* were used as endogenous controls for normalization. The relative gene expression was calculated by the 2-^ΔΔCt^ method in biological triplicates (*n* = 3). The bars represent standard errors and the asterisks (*) indicate statistical significance determined by the Student’s t-test (*P* ≤ 0.05).

**Additional file 2: Table S1.** *Cis*-regulatory elements related to drought revealed in the p*GmRD26* soybean promoter and the *A. thaliana* promoter *RD29* (*p*-value of 0.05).

**Additional file 3: Table S2.** Primer sequences used in the qRT-PCR analysis.

## Data Availability

The datasets supporting the conclusions of this article are included within the article and its additional files. All plant materials were obtained from Embrapa Genetic Resources and Biotechnology. Brasília- Brazil.

## Abbreviations

ABA: Abscisic acid
ABRE: ABA-responsive element
*AtRD29*: *Arabidopsis thaliana* responsive to desiccation 29
DREB: Dehydration-responsive element binding
MYB: Myeloblastosis
MYC: Myelocytomatosis
NAC: Nitrogen assimilation control
PEG: Polyethylene glycol
p*GmRD26*: *Glycine max* responsive to desiccation 26 promoter
TFs: Transcription factors
