# Supplementary material for: Identification and characterization of the GmRD26 soybean promoter in response to abiotic stresses: potential tool for biotechnological application
Source: BMC Biotechnol. 2019 Nov 20;19:79. doi: 10.1186/s12896-019-0561-3 (PMC6865010; doi:10.1186/s12896-019-0561-3)
Supplement: Supplementary file 1 — Additional file 1: Figure S1. GmNAC085 expression profile in soybean (Williams 82) under multiple stresses. To determine the gene expression profile of the GmNAC085 gene, the soybean seedlings were submitted to different stress conditions (ABA, PEG, AS, Tun, and drought), and the gene expression were analyzed in leaves and roots by qRT-PCR. The fold change values were calculated in comparison of plants treated with untreated plants (0 h). CYP2 and ELF1A were used as endogenous controls for normalization. The relative gene expression was calculated by the 2-ΔΔCt method in biological triplicates (n = 3). The bars represent standard errors and the asterisks (*) indicate statistical significance determined by the Student’s t-test (P ≤ 0.05). [file 12896_2019_561_MOESM1_ESM.docx]

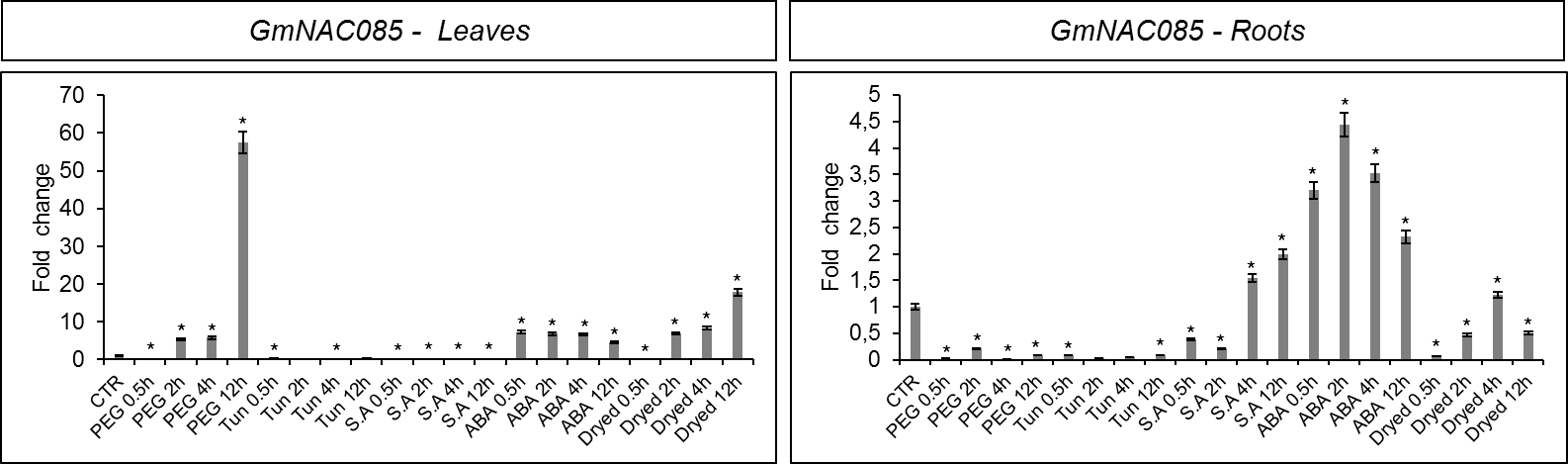


**Additional file 1:** **Figure S1**. *Gm*NAC085 expression profile in soybean (Williams 82) under different stress. To determine the gene expression profile of the *Gm*NAC085 gene, the soybean seedlings were submitted to different stress conditions (ABA, PEG, AS, Tun and drought), and the gene expression in leaves and roots was analyzed by qRT-PCR. The fold change values were calculated in comparison of plants treated with untreated plants (0h). CYP2 and ELF1A were used as endogenous controls for normalization. The relative gene expression was calculated by the 2-ΔΔCt method in biological triplicates (n = 3). The error bars represent ± standard errors and the asterisks (*) indicate statistical significance determined by the Student’s t-test (P≤0.05).
