# Supplementary material for: Identification and characterization of the GmRD26 soybean promoter in response to abiotic stresses: potential tool for biotechnological application
Source: BMC Biotechnol. 2019 Nov 20;19:79. doi: 10.1186/s12896-019-0561-3 (PMC6865010; doi:10.1186/s12896-019-0561-3)
Supplement: Supplementary file 2 — Additional file 2: Table S1. Cis-regulatory elements related to drought revealed in the pGmRD26 soybean promoter and the A. thaliana promoter RD29 (p-value of 0.05). [file 12896_2019_561_MOESM2_ESM.docx]

**Additional file 2: Table S1.** *Cis*-regulatory elements related to drought revealed in the p*Gm*RD26 soybean promoter and the *A. thaliana* promoter RD29 (p-value of 0.05 )

| ***Cis*-regulatory element** | **Core sequence** | **Number of**  ***Cis*-regulatory elements** | | **Description** |
| --- | --- | --- | --- | --- |
|  |  | ***Gm*RD26** | ***At*RD29A** |  |
| ACGTATERD1 | ACGT | 5 | 5 | Dehydration |
| DREDR1ATRD29AB | TACCGACAT | 0 | 2 | Dehydration; high salt |
| DRECRTCOREAT | RCCGAC | 0 | 4 | Drought |
| DRE2COREZMRAB17 | ACCGAC | 3 | 3 | Dehydration and ABA |
| MYCCONSENSUSAT | CANNTG | 4 | 2 | Dehydration, ABA and Cold |
| ACGTABREMOTIFA2OSEM | ACGTGKC | 3 | 1 | Dehydration and ABA |
| MYB2CONSENSUSAT | YAACKG | 1 | 0 | Dehydration and ABA |
| ABREZMRAB28 | CCACGTGG | 1 | 0 | ABA responsive |
| ABREATCONSENSUS | YACGTGGC | 2 | 0 | ABA responsive |
| MYBCORE | CNGTTR | 4 | 0 | Dehydration and ABA |
| MYB1AT | WAACCA | 1 | 1 | Dehydration and ABA |
| MYB2AT | TAACTG | 1 | 0 | Dehydration |
| MYCATERD1 | CATGTG | 0 | 1 | Dehydration |
| MYCATRD22 | CACATG | 0 | 1 | Dehydration; ABA |
| LTRECOREATCOR15 | CCGAC | 0 | 4 | Cold; drought, ABA |
| G-box | CACGTG | 4 | 0 | Dehydration, high salinity, ABA |
| EBOXBNNAPA | CANNTG | 4 | 2 | ABA responsive |
| DPBFCOREDCDC3 | ACACNNG | 3 | 2 | ABA responsive |
| ABRERATCAL | MACGYGB | 4 | 1 | ABA responsive |
