# Supplementary material for: Identification and characterization of the GmRD26 soybean promoter in response to abiotic stresses: potential tool for biotechnological application
Source: BMC Biotechnol. 2019 Nov 20;19:79. doi: 10.1186/s12896-019-0561-3 (PMC6865010; doi:10.1186/s12896-019-0561-3)
Supplement: Supplementary file 3 — Additional file 3: Table S2. Primer sequences used in the qRT-PCR analysis. [file 12896_2019_561_MOESM3_ESM.docx]

**Additional file 3: Table S2** - Primer sequences used in the qRT-PCR analysis

| **Gene name** | **Forward primer sequence [5´-3´]** | **Reverse primer sequence [5´-3´]** |
| --- | --- | --- |
| Glyma.06G248900 | ATTCTTCCCGCAAACACAAC | CATTTATCTCCGGCAACGAT |
| CYP2 | CGGGACCAGTGTGCTTCTTCA | CCCCTCCACTACAAAGGCTCG |
| ELF1A | GACCTTCTTCGTTTCTCGCA | CGAACCTCTCAATCACACGC |
| GUS | TTGGGCAGGCCAGCGTATCGT | ATCACGCAGTTCAACGCTGAC |
| ACT2 | TTTCACTATATGCCAGTGGTCG | CTTCGTAGATCGGGACAGTGTG |
| GAPDH | GGTCATGGGAGATGACATGGTC | CAGGGTTTGTCTCGCAAAAATC |
